# The influence of warming and biotic interactions on the potential for range expansion of native and nonnative species

**DOI:** 10.1093/aobpla/plaa040

**Published:** 2020-08-18

**Authors:** Betsy von Holle, Sören E Weber, David M Nickerson

**Affiliations:** 1 Department of Biology, University of Central Florida, and Division of Environmental Biology, National Science Foundation, 2415 Eisenhower Avenue Alexandria, VA, USA; 2 Institut für Evolutionsbiologie und Umweltwissenschaften, University of Zurich, Winterthurerstrasse 190, Zürich, Switzerland; 3 Department of Statistics, University of Central Florida, 4000 Central Florida Blvd, Orlando, FL, USA

**Keywords:** Climate change, enemy release, *Eugenia*, invasion, plant–soil microbe interactions, range expansion

## Abstract

Plant species ranges are expected to shift in response to climate change, however, it is unclear how species interactions will affect range shifts. Because of the potential for enemy release of invasive nonnative plant species from species-specific soil pathogens, invasive plants may be able to shift ranges more readily than native plant species. Additionally, changing climatic conditions may alter soil microbial functioning, affecting plant–microbe interactions. We evaluated the effects of site, plant–soil microbe interactions, altered climate, and their interactions on the growth and germination of three congeneric shrub species, two native to southern and central Florida (*Eugenia foetida* and *E. axillaris*), and one nonnative invasive from south America (*E. uniflora*). We measured germination and biomass for these plant species in growth chambers grown under live and sterile soils from two sites within their current range, and one site in their expected range, simulating current (2010) and predicted future (2050) spring growing season temperatures in the new range. Soil microbes (microscopic bacteria, fungi, viruses and other organisms) had a net negative effect on the invasive plant, *E. uniflora*, across all sites and temperature treatments. This negative response to soil microbes suggests that *E. uniflora*’s invasive success and potential for range expansion are due to other contributing factors, e.g. higher germination and growth relative to native *Eugenia*. The effect of soil microbes on the native species depended on the geographic provenance of the microbes, and this may influence range expansion of these native species.

## Teaser

Species ranges are expected to shift in response to climate change; however, it is unclear how species interactions will affect range shifts. Our study demonstrates that, in the predicted future range, plant–soil microbial interactions appeared as a form of biotic resistance to one native and one nonnative shrub species, however soil microbes facilitated a native species in the future range, under warmer temperatures. Plant–soil microbe interactions for the native congeners depended on the geographic provenance of the soils, which may affect their ability to expand their ranges.

## Introduction

Understanding species responses to global change will help predict shifts in species distributions as well as aid in conservation planning and management. Climate change varies around the world and concomitant ecological responses are likely to differ by region (Walther *et al.* 2002). During the past century, average annual global temperatures for land and ocean surfaces have increased at a rate near 0.6 °C/century; however, the trend has been three times greater since 1976, with some of the largest increases in temperature occurring in the high latitudes (NOAA Satellite and Information Service 2009). As the climate changes, most species have been shifting their ranges poleward, or upward on mountains (Parmesan and Yohe 2003); however, species vary in their ability to shift their ranges (Sorte *et al.* 2010). Growing evidence suggests that biotic interactions play key roles in species response to climate change (Guo *et al.* 2013). Specifically, the study of above- and below-ground biotic interactions are important to consider in relation to plant range expansion with climate change (van der Putten *et al.* 2016). In a literature review of over 600 papers, Tylianakis *et al.* (2008) revealed that climate change influences virtually every type of biotic interaction, yet biotic interactions are rarely incorporated into models of organismal response to climate change (Gilman *et al.* 2010). Gilman *et al*. (2010) stressed the importance of assessing the potential effects of novel interactions precipitated by climate change, suggesting that assessing species demographic responses to interactions with species beyond current range boundaries would improve our ability to understand species range-expanding capacities.

Soil microbial communities comprise symbionts and decomposers, which generally results in positive plant soil feedbacks (PSF) (Ehrenfeld *et al.* 2005) as well as soil pathogens, which result in negative PSF (Reinhart and Callaway 2006; Morris *et al.* 2007; Kulmatiski *et al.* 2008). Nonnative plant species may be able to expand their ranges more readily than native species because they may have fewer enemies (Klironomos 2002; Hawkes *et al.* 2009) when compared with their native range [‘enemy release hypothesis’ (Keane and Crawley 2002)], maintain the ability to form positive mycorrhizal interactions in their new ranges (Callaway *et al.* 2011), receive greater positive impacts in the new range than the native range [‘enhanced mutualism hypothesis’ (Reinhart and Callaway 2006; Callaway *et al.* 2011)], reduce local symbionts (Stinson *et al.* 2006; Vogelsang and Bever 2009), alter secondary metabolites (Wardle *et al.* 1998) and nutrient dynamics in new ranges (Kourtev *et al.* 1998; von Holle *et al.* 2013). Specifically, nonnative plant species may be able to expand their ranges more readily than native species because they encounter fewer co-evolved root pathogens, the absence of which may allow greater colonization by generalist mycorrhizal fungi in their new ranges, benefitting invasive plants (Beckstead and Parker 2003; van Grunsven *et al.* 2007; Hawkes *et al.* 2010). Additionally, soil biota may not be able to shift their ranges as quickly as plant species can (Berg *et al.* 2010), which may have strong effects on the potential for both native and nonnative plant range expansion and community composition (van Grunsven *et al.* 2007; Engelkes *et al.* 2008; van Grunsven *et al.* 2010).

Warming is expected to benefit plant–mycorrhizal interactions and nutrient cycling, which will affect plant nutritional status as well as aboveground plant–insect interactions (Koricheva *et al.* 2009). Microbial responses to warming can be short-lived (Balser *et al.* 2006), potentially due to changes in microbial community composition, substrate availability, altered litter quality, or physiological adjustments of the soil biota (Pritchard 2011). Soil warming has been found to promote soil microbial activity, net nitrification rates, P and N mineralization rates and total respiration in soil (Andresen *et al.* 2010). In a meta-analysis of mycorrhizal response to global change, warming increased mycorrhizal fungal abundance in 63 % of the studies, whereas mycorrhizal activity decreased in 71 % of the studies (Mohan *et al.* 2014). It is expected that the activity and abundance of soil pathogens will increase with rising temperatures because their life cycles will be shortened (Bragazza *et al.* 2013). While there is strong evidence for an increase in aboveground plant pathogens with warming, little is known about the effects of warming on below-ground pathogens and their effects on natural plant populations (Van der Putten *et al.* 2010).

Our research addressed the question of whether nonnative species will be better potential range-expanders than native species because of their propensity to have less negative plant–soil microbial interactions, or a net positive interactions with microbes, after invading a new area (Diez *et al.* 2010). We hypothesized that the ability of nonnative species to expand their range would be greater than native species, because of their ability to form positive generalist associations with soil biota and the likelihood that they will escape species-specific soil enemies (Hawkes 2007). Further, we predicted that nonnative species would have net positive associations with soil biota, and that these positive associations will increase outside their range, with plant–microbe interactions more positive in their new range than in their current range (Callaway and Aschehoug 2000; Diez *et al.* 2010). Additionally, we assessed whether plant–soil microbe interactions for a nonnative species are affected by climate change. We expected that warming would enhance nonnative plant growth, and that the effect of warming on plant growth would be enhanced by plant–microbe interactions because there would be fewer negative interactions from co-evolved soil pathogens outside of its current range. We expected that native plant species would have negative plant–microbe interactions in their current range, but that these would switch to positive plant–microbe interactions outside of their range, because of a lack of coevolved pathogens in the new range.

## Methods

### Study species

Closely related native and nonnative species were used for this experiment to control for species responses that are attributable to phylogeny and isolate the response of plant origin (native, nonnative) to climate change and soil biota. We chose species from *Eugenia,* in the Myrtaceae family, because there are closely related native and nonnative species of the same genus and functional group which co-occur in subtropical hammock habitat of Florida (Liu *et al.* 2007). All three species are relatively abundant throughout Florida (Wunderlin *et al.* 1996). These small tree and shrub species are found in subtropical habitats in Florida, central and South America and the Caribbean. *Eugenia uniflora*, or Surinam cherry, is native to Brazil and has been introduced to much of South America outside of Brazil, in addition to Asia, Australasia-Pacific Region, Europe and North America (Wunderlin *et al.* 1996; ISSG 2016). *E. uniflora* associates with arbuscular mycorrhizal fungi (Zangaro *et al.* 2005). *E. uniflora* was introduced to Florida as an ornamental and for its edible fruit prior to 1931, and has been widely planted in central and south Florida, especially for hedges (Langeland *et al.* 2008). *E. uniflora* has a high impact on ecological communities (FLEPPC 2017), is able to invade upland habitat, and is located south of the freeze line in Florida. *E. uniflora* is considered Category I, as designated by the Florida Exotic Pest Plant Council (FLEPPC 2017), which is a species that causes large ecological damage through the displacement of native species, changing community structures or ecological functions, or hybridizing with natives.

### Growth chamber experiment set up

Changes in growth and germination of our three *Eugenia* study species was monitored in pots placed in growth chambers, using upland hammock soils from their current range in Florida and from their potential climatically induced expanded range. Central Florida is the current northern limit of *Eugenia* species in Florida (Wunderlin and Hansen 2003), and so we chose a site with hammock habitats that was north and well outside of their current range, as predicted by the poleward expansion of species (Parmesan and Yohe 2003). Future temperature conditions of the northern site were estimated with a Low, B1 emission scenario; for a range of SRES emissions scenarios, and using global climate projections from the Fourth IPCC Assessment (IPCC 2007; Girvetz *et al.* 2009). Pots were placed in growth chambers where diurnal variation in daylength and temperatures were simulated, with the high and low daily temperatures determined by the average daily maximum and minimum temperatures for the month of May in Jacksonville, FL (Florida Climate Center, Center for Ocean-Atmospheric Prediction Studies), the northernmost site from where soil was collected. The pots experienced environmental conditions simulated for current (2010) and future (2050) conditions, with 10 h of light per day and 30/17 °C and 31/18 °C and day/night temperatures ("Table 1A and B).

**Table 1.** Experimental design for growth chambers *Eugenia* species. Treatment type is in bold and numbers indicate the number of pots within each treatment.

		A. *E. axillaris* and *foetida*											
		**Climate**											
		Current						Future					
		**Chamber**											
		A						B					
		**Species**						**Species**					
		*axillaris*			*foetida*			*axillaris*			*foetida*		
		**Site**			**Site**			**Site**			**Site**		
		South	Central	North	South	Central	North	South	Central	North	South	Central	North
Soil	nonsterile	9	9	9	9	9	9	9	9	9	9	9	9
	sterile	8	8	8	8	9	9	7	9	9	7	9	9
		B. *E. uniflora*											
		**Climate**											
		Current						Future					
		**Chamber**						**Chamber**					
		F			E			D			C		
		**Site**			**Site**			**Site**			**Site**		
		South	Central	North	South	Central	North	South	Central	North	South	Central	North
Soil	nonsterile	7	—	—	—	7	7	7	—	—	—	7	7
	sterile	7	7	—	—	—	7	—	7	7	7	—	—

### Seed sampling

Seeds were haphazardly collected from populations located within Hugh Taylor Birch State Park, in south Florida, for the one nonnative and two native study species. Seeds were collected for each species at the peak of seed production for their species. Seeds for the native species were collected on 17 December 2011, and seeds for the nonnative *Eugenia* species were collected on 28 April 2012. The fruit covering from each seed was removed by hand and the seeds were surface sterilized in 5 % bleach solution for 15 min, and washed with de-ionized water, prior to planting.

### Soil sampling

Soil was collected from three hammock habitat sites within each of the central, south and north Florida sites. Soil biota was collected in the form of fresh field-collected soil from one of two sources: the current home range [Central Florida (Cape Canaveral, FL), South Florida (Hugh Taylor Birch State Park)] or within the projected new range [North Florida (Timucuan Ecological and Historic Preserve, Florida)]. Soils were collected from all three Florida source regions within 1 week prior of the potting date, to ensure viability of the soil microbiota. In the south and central Florida sites, we collected soil from hammock habitats within natural areas which were at least 20 m from *Eugenia* shrubs or seedlings. In the north Florida site, we collected soil from randomly placed transects (using random point generator feature of ArcMap, ESRI, Redlands, CA) within hammock habitats. Within each of these three sites, two, 10-m transects were laid within hammock habitat, at least 5 m away from roads. Every 2 m, 10 cm deep soil samples were collected and placed into a Ziploc bag. The two, 10 m transects were parallel and at least 10 m apart. Soil samples were combined within each site, sieved to 2 mm, and added to the pots within 1 week of collection (as in Hawkes *et al.* 2011). We pooled soils within each site to provide a soil inoculum treatment representing all possible soil microbes in that site and the average density found within that site (Cahill *et al.* 2017), which is a common treatment used to understand the effect of soil microbes on plant germination and growth (Grman and Suding 2010; Lau and Lennon 2011; Farrer and Suding 2016); however, this method can artificially decrease variation in plant–microbe interactions (Reinhart and Rinella 2016; Rinella and Reinhart 2017, 2018). While variation in plant–microbe interactions is decreased with pooling samples, this method of soil pooling is preferable when the objective is to understand if the average pathogen density found in each of two regions differentially effects plant growth (Cahill *et al.* 2017). The soil biota treatment is one of several treatments, where we evaluate plant–microbial interactions in relation to those treatments.

The soil biota treatment was fresh field-collected soil from each of the central, south, and north Florida sites. For the control treatment, we sterilized half of these field-collected soils from the current and new ranges. The sterile soil inoculum was autoclaved three times, and we mixed the soil in between autoclave events, to ensure sterilization of the soils. The soil biota and sterile control inocula comprised 5 % of the mass of the pot, to ensure sufficient inoculation of the soil biota to the pot while also maintaining the same nutrient conditions and soil characteristics across all treatments (as in Reinhart and Callaway 2006).

For each species, eight seeds were planted into a minimum of seven, sterile replicate pots (4 × 4 × 6″) filled with sterile potting mix (MetroMix 366 sterile potting soil) and one of two soil inoculum treatments (sterile, nonsterile), two temperature treatments 2010 (e.g. ‘current’) and 2050 (e.g. ‘future’ temperature conditions at our northernmost site) and three site treatments (south, central and north), for a total of 86 pots for the nonnative species and 105 pots each for the native species. Eight to nine replicate pots per treatment were made for the native species (Table 1a), and seven replicate pots were made per treatment for the nonnative species (Table 1b), as the native species have lower germination rates, relative to the nonnative *Eugenia* species (Stricker and Stiling 2013). The potting dates were 27 January 2012, for the native species, and 9 May 2012, for the nonnative species, in accord with their fruiting phenology and when the seeds were collected. After germination, pots were kept in a growth chamber for the next 12 weeks, to assess growth. They were watered daily with equal amounts of water, approximately 15–20 mL. Pots were rotated daily within the growth chamber, to control for positional effects. Care was taken to ensure that the soil biota were not cross-contaminated between pots by using sterile techniques. Germination was monitored weekly until after the appearance of the first germinant, at which point monitoring occurred daily. Daily monitoring ceased after the pots were monitored daily for 2 weeks with no new germination. Two weeks after germination ceased for each species, we selected a maximum of four seedlings to remain, and removed all other seedlings from the pot, taking care not to disturb the soil. Twelve weeks following initiation of germination, the remaining plants were harvested for total above and below-ground biomass. Shoots were cut at ground level and oven-dried separately in paper bags at 60 °C for 2 days. The roots were carefully washed to remove soil particles and also oven-dried at 60 °C in paper bags. After drying, shoots and roots were weighed with a precision balance to determine dry weight.

### Statistical design and analyses

Our objective was to determine differences in closely related native and nonnative species responses to soil microbiota under conditions of range expansion and climate change. The difference in plant performance between the pots that had the living soil inoculum to those of the sterile controls was due to differences in plant response to soil biota. Additionally, the difference in native and nonnative plant performance in its home soils versus its expanded range soils was used to assess differences in plant–soil microbe interactions between home and away sites. Last, differences in plant performance between temperature treatments can be used to assess differences in plant–microbe interactions under climate change.

### Nonnative *Eugenia*

The experimental design for the nonnative *E. uniflora* was an incomplete split-plot design with climate as the main plot treatment, growth chambers nested within climate as the main plots and site and soil as subplot treatments. Total biomass was analysed with a generalized linear mixed model (Wolfinger and O’Connell 1993) using SAS/GLIMMIX. Climate, site and soil were fixed effects and growth chamber within climate was a random effect. The distribution was specified as lognormal and the link was specified as identity. The estimation method used was the default RSPL (residual pseudo-likelihood with expansion locus the vector of random effects solutions). With the pattern of missing combinations of climate, site and soil, it was determined that all main effects and interactions, except for the three-way interaction, could be tested for significance. Any significant effects were followed up by appropriate pairwise comparisons with suitable sequential Bonferroni corrections (Holm 1979).

### Native *Eugenia*

We evaluated current and future climate treatments on the total biomass of *E. axillaris* and *E. foetida* separately, as opposed to together, as was done for the nonnative *E. uniflora*. Within each level of climate, we had a complete three-way factorial experiment with all combinations of species, site and soil. Here, we had no replication (multiple growth chambers) for each level of climate. Consequently, we could not directly test for climate in combination with the other factors.

A large number of total biomass values were zero due to the lack of germination of *E. axillaris* and *E. foetida*. Consequently, a finite mixture model (McLachlan and Peel 2000) was chosen to analyse the data. A finite mixture model is a weighted average of two or more models. We used a two-point mixture distribution where the distribution was degenerate at zero in the case of no germination and the other was a lognormal distribution, conditional on germination. Both individual models are generalized linear models. The mixing probabilities were the probability of no germination for the degenerate distribution and the probability of germination for the lognormal distribution.

The analysis was conducted in two steps, using a hurdle model (Cameron and Trivedi 1998). Each step included a generalized linear mixed model using SAS/GLIMMIX with species, site and soil as fixed effects. Additionally, the estimation method used was LAPLACE (approximates the marginal likelihood by using the Laplace method) since it more closely resembled the results of SAS/FMM using dual quasi-Newton optimization. First, the probability of germination was separately analysed for each level of climate. The distribution was specified as binomial and the link was specified as logit. The second step was the analysis of total biomass conditional on germination. In other words, for those seeds that did germinate, i.e. those that clear the hurdle, the total biomass can then be affected by the treatments. This is tantamount to eliminating all observations where there was no germination (zero biomass) and analysing the remaining responses. The distribution was binomial with a logit link. Any significant effects were followed up by appropriate pairwise comparisons with suitable sequential Bonferroni corrections (Holm 1979).

## Results

### Germination of nonnative *Eugenia*

*E. uniflora* had high germination success, with 99 % of the pots having at least one germinant, across all treatments. There were no significant differences in germination within site, temperature or soil treatments, according to the generalized linear mixed model of germination by pot [see Supporting Information—**Table S1**].

### Nonnative *Eugenia*–microbe interactions

**Figure 1.**
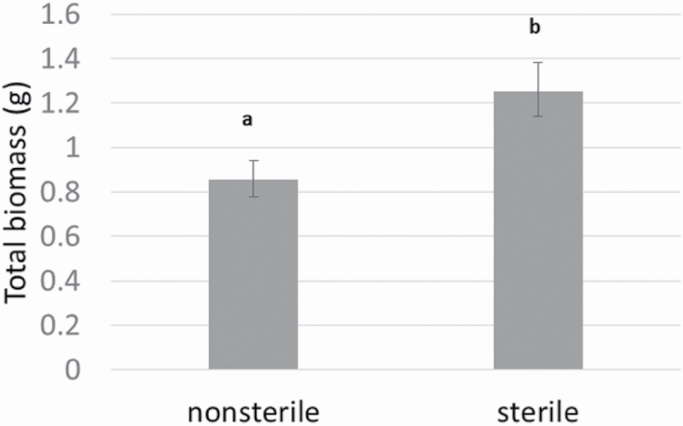
Total biomass, at the end of the experiment of *Eugenia uniflora*. Weight of attached seeds was removed from the total biomass estimation. Statistically significant differences are indicated by different letters (*t*-value: -5.45, df = 71, *P* < 0.0001).

The nonnative *E. uniflora* had significantly lower biomass when grown in soils containing soil microbes, indicative of a negative plant–soil microbe interaction for this species (Fig. 1), and this held across all sites and temperature conditions (Table 2). Soil biota from native hammock habitats have a negative effect on the growth of nonnative *E. uniflora* (Fig. 1), regardless of site and temperature conditions. In the generalized linear mixed model, the ‘site*soil’ term was not significant, suggesting that a negative plant–soil microbe interaction is consistent across sites, including outside of its current range, contrary to our hypothesis that plant–microbe interactions would be positive outside of the current range of this species (Table 2).

**Table 2.** *Eugenia uniflora* biomass effect tests for climate (current, future), site (north, central and south) and soil (nonsterile, sterile).

Effect	Num DF	Den DF	*F* Value	Pr > *F*
Climate	1	2	0.36	0.6111
Site	2	71	1.21	0.3028
Climate × site	2	71	6.37	0.0029***
Soil	1	71	29.69	<0.0001***
Climate × soil	1	71	0.04	0.8451
Site × soil	2	71	0.64	0.5299

***Significant at 1% level.

### The effects of temperature and site on nonnative *Eugenia*

**Figure 2.**
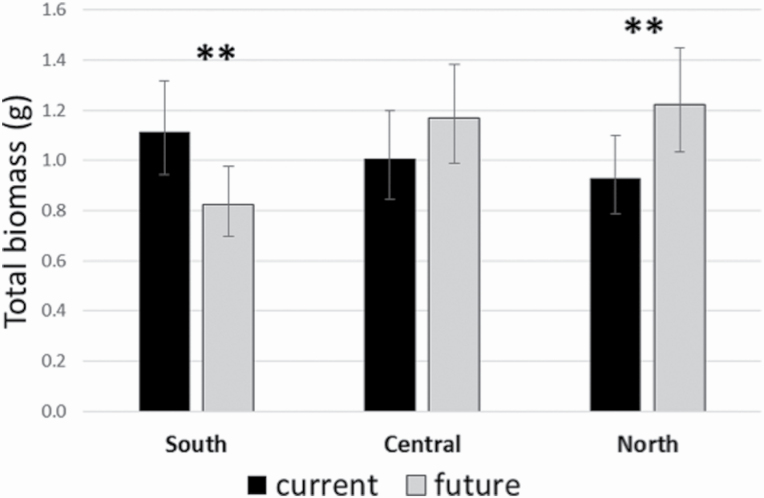
Total biomass of *Eugenia uniflora* grown in soils from each site, grown under current and future temperatures. Statistically significant differences (at the 5% level) between current and future temperatures at each site are indicated by a double asterisks (**).

The climate*soil term was not significant in the generalized linear mixed model, suggesting that the net negative effect of the soil biota on nonnative *Eugenia* growth is similar for both the future and current temperature treatments. Total biomass of the invasive species *E. uniflora* depended on the site and the climatic conditions under which it was grown, as indicated by the significant climate by site interaction (Table 2). Three separate pair-wise comparisons of current to future temperature were made for each level of site. *P*-values of current versus future temperature at the following sites were: South (0.0144), Central (0.2226) and North (0.0248). Plants grown in soil from the northern site had significantly greater biomass when grown in warmer temperatures than in current temperatures (Fig. 2). This relationship switched in the southern site, where total biomass of this species was greatest under current temperatures, when compared with future, warmer temperatures (Fig. 2). Under current temperatures, there were no statistically significant differences of *E. uniflora* between sites [*P*-values: South versus Central (0.4026), South versus North (0.1326) and Central versus North (0.5181)]. When grown under warmer temperatures, *E. uniflora* had significantly lower biomass in soils from the southern-most site, when compared with the central and northern sites [*P*-values: South versus Central (0.0049**), South versus North (0.0016***) and Central versus North (0.7079)].

### Germination and biomass of native *Eugenia*

There were no significant differences in germination between native species, site or soil for either level of climate [****see Supporting Information—**Table S2a and b**]. Eighteen percent of *E. axillaris*-planted pots had at least one seedling germinate, and the pot-level germination rate of *E. foetida* was 71 %.

Total biomass conditional on germination was separately analysed for each level of climate with a generalized linear mixed model. When grown under current temperatures, there were no significant effects of native species, site or soil on total biomass [**see Supporting Information—Table S3**]. Under future climate conditions, there was a significant main effect for site, a significant two-way interaction between species and site, and more importantly, a significant three-way interaction between species, site and soil (Table 3).

**Table 3.** Biomass effect tests for native *Eugenia* species (*axillaris, foetida*), site (north, central, and south) and soil (nonsterile, sterile) at climate = future, conditional on germination.

Effect	Numerator df	Denominator df	F	*P*-value
Species	1	38	0.92	0.3442
Site	2	38	4.98	0.0120**
Species × site	2	38	3.72	0.0333**
Soil	1	38	0.06	0.8046
Species × soil	1	38	0.90	0.3480
Site × soil	2	38	1.87	0.1679
Species × site × soil	2	38	5.49	0.0080***

***Significant at 1% level.

**Significant at 5% level.

**Figure 3.**
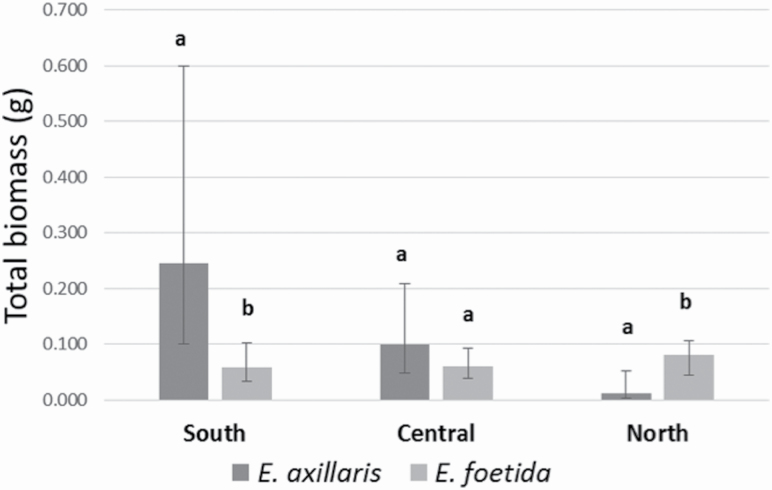
Total biomass of natives *Eugenia axillaris* and *E. foetida*, for nonsterile soil, and future climate. Statistically significant differences (at the 5% level) are indicated by different letters.

Total biomass of the native *E. axillaris* grown in nonsterile soils were higher when grown in soils from the southern site soils than when grown in soils from the northern site (Fig. 3), suggesting that soil microbes from the southern sites benefit *E. axillaris* more than soil microbes from the northern sites, contrary to our hypothesis that plant–microbe interactions would be positive outside of its current range, when compared with within its current range. When this species was grown in sterilized soil from the different sites, total biomass was not significantly different between the sites.

In the northernmost site, outside of their current range and under future temperature conditions, *E. axillaris* had significantly lower biomass than *E. foetida* when grown in nonsterile soils (Fig. 3). This suggests that *E. foetida* benefits more from the soil microbial community in the new, northern range, than *E. axillaris*. As indicated by the significant three-way interaction between species, site and soil (Table 3), these relationships switched in the southernmost site, where *E. axillaris* had significantly greater biomass when grown in nonsterile soils, when compared with *E. foetida*. This suggests that in the southernmost sites where they currently co-occur, *E. axillaris* is benefitted by the soil microbes more than *E. foetida*, under future climatic conditions (Fig. 3).

## Discussion

### Nonnative *Eugenia*–microbe interactions

Soil biota from native hammock habitats have a negative effect on the growth of nonnative *E. uniflora,* irrespective of site and temperature conditions. Our finding of a negative plant–soil microbe interaction across all sites, including outside of its current range, is contrary to our hypothesis that plant–microbe interactions would be positive for this nonnative species, especially outside of the current range of this species. Pathogens tend to cluster phylogenetically (Agrawal and Kotanen 2003), and could ‘hop’ from native to nonnative hosts with relative ease (Parker and Gilbert 2004), which may be especially true of our nonnative study species, which co-occurs with native congeners in the hammock habitat of our southern and central sites (Liu *et al.* 2007; Stricker and Stiling 2013), including our two native study species. Research focusing on PSF of nonnative species which invade habitats with resident congeners and have a higher shared evolutionary history differ substantially from studies which simply compare PSF between native and nonnative species, regardless of their evolutionary history (Suding *et al.* 2013). For example, in Europe, the fungal communities associated with the introduced lodgepole pine (*Pinus contorta*) comprised those species associated with the local native Scots pine, *P. sylvestris*; however, in South America where the most closely related native species is in the *Nothofagus* genus, the fungal communities of this introduced species comprised those found from its native range (Gundale *et al.* 2016).

For invasive plants, enemy release has been estimated to attenuate over the course of approximately 200 years (Hawkes 2007; Diez *et al.* 2010; Lankau 2011), likely due to an increasing chance of soil pathogens arriving from their home range (Hallett 2006). While it is possible that *E. uniflora* experienced enemy release upon introduction to Florida in 1931, it seems unlikely that enemy release for this species would attenuate more than twice as rapidly as enemy release in other studies.

Negative plant–microbe interactions for the nonnative *E. uniflora* indicates a form of biotic resistance to invasion (Simberloff and Gibbons 2004; Kardol *et al.* 2007), rather than facilitation by the existing soil biota (Richardson *et al.* 2000), or enemy release from species-specific soil pathogens (Klironomos 2002). A meta-analysis of paired native and nonnative PSF studies revealed that while native species generally have positive PSFs, nonnative species may have either positive or negative PSFs (Suding *et al.* 2013).

### The effects of temperature and site on nonnative *Eugenia*

We explored how plant–soil microbe interactions varied across current and future temperatures, as well as current and future ranges for the nonnative study species. We expected that warming would enhance nonnative plant growth, and that the effect of warming on plant growth would be enhanced by plant–microbe interactions because microbial activity increases with temperature. While warming significantly enhanced growth of the nonnative plant species grown in central and northern soils, the opposite was found for plants grown in southern soils. Given that the interaction between climate and soil, and site and soil were not significant, these results do not necessarily reflect plant–soil microbe interactions. The increased biomass of the nonnative species in central and northern soils, under warmer conditions, may reflect some other aspect of the soils from the different parts of the range. Additionally, we expected that there would be fewer negative interactions from co-evolved soil pathogens outside of its current range and a net positive plant–soil microbe interaction in the new range, which we did not find. In accord with our study, conducted in growth chambers with a difference of 1 °C, a greenhouse study comparing plant–microbe interactions of native and nonnative species in the Netherlands found a temperature difference of 5 °C did not influence net plant–microbe interactions of either the native or closely related nonnative species (van Grunsven *et al.* 2010). In a meta-analysis of natural area responses to global change drivers, mycorrhizal abundances have been found to increase under warmer conditions, however mycorrhizal activity has been found to decrease under warmer conditions (Mohan *et al.* 2014), which may explain this pattern. Given that the plant–microbe interactions for this invasive species did not change under different temperature conditions, it is unlikely that warming significantly increased below-ground pathogens (Van der Putten *et al.* 2010), relative to soil microbiota with positive effects.

### Native *Eugenia*–microbe interactions

We expected that native plant species would have negative plant–microbe interactions in their current range, but positive plant–microbe interactions outside of their current range, because of a lack of co-evolved pathogens in the new range. We found evidence that the native *E. axillaris* benefited from soils from their current range relative to *E. foetida*, however this relationship switched in the new range, under future climatic conditions. Given that there is a strong positive relationship between plant–soil feedback and the abundance of plants in the field (Klironomos 2002), we expect that the abundance of *E. axillaris* would be greater than *E. foetida* in its current range, however, given the trend of a linear decrease of *E. axillaris* biomass as it was grown in soils increasingly outside of its range, from south to north (Fig. 3), it may be less likely to expand its range northward than *E. foetida* would under warmer temperatures.

Early successional forest trees have been documented to be dominated by soil pathogens and root herbivores (Packer and Clay 2000), and so our study species may be more likely to have negative plant–soil microbe interactions than other functional groups. Further, arbuscular mycorrhizal fungal tree systems tend to have negative plant–soil microbe interactions, in general (Bennett *et al.* 2017). It is also likely that the degree to which the plant–soil microbe interactions manifested for our species, grown in growth chambers, was greater than what would be observed in the field (Schittko *et al.* 2016).

### Germination and biomass of native and nonnative *Eugenia*

Our research addressed the question of whether nonnative species will be better potential range-expanders than native species because of their propensity to have less negative plant–soil microbe interactions, or a net positive plant–microbe interactions, after invading a new area (Diez *et al.* 2010). The germination success of the nonnative species, *E. uniflora*, across all temperature and soil treatments, underscores the invasive potential of this nonnative species (Liu *et al.* 2006; Langeland *et al.* 2008; Stricker and Stiling 2013). Invasiveness may depend on the physiology of the invaders, and their ‘pre-adaptations’ (Rejmanek and Richardson 1996; Parker and Hay 2005). In particular, Rejmanek and Richardson (1996) found that the best measures of invasiveness of *Pinus* species were factors of mean seed mass, minimum juvenile period and the average time between large seed-producing events, with the most invasive species having lower average seed mass, shorter juvenile periods and short times between when large crops of seeds are released. It is relatively intuitive that these characters confer a greater degree of invasiveness to a given species. If a species reaches maturity quickly, devotes less energy to each individual seed, and has short intervals between seed crops, this may quickly result in the outnumbering of other co-occurring species, especially in disturbed, open habitats. Our invasive study species had extremely high levels of germination and accrued a large amount of biomass in the 12 weeks following germination, relative to the native congeners.

The lack of significant differences in germination between native species, site or soil for either level of climate may be due to the low power associated with the growth chamber experiments for the two native species. Another possibility is there are seeds that will not germinate regardless of species, site or soil treatments, which would indicate that a hurdle model is appropriate to use (Cameron and Trivedi 1998). For those seeds that do germinate, i.e. those that clear the hurdle, total biomass can then be affected by the treatments. Given the low germination success of the native species, especially *E. axillaris*, future experiments should increase the number of replicates of the native species to increase the ability to detect differences in germination.

Plant herbivory for our nonnative study species was found to be higher than that for its native, co-occurring congeners in a separate study conducted in our southern-most site (Stricker and Stiling 2012), largely due to a recently introduced nonnative weevil from Sri Lanka, *Myllocerus undatus*. Interestingly, in a study 4 years prior on the same species and at the same site, the enemy release hypothesis was supported because significantly more specialized (oligophagous and endophagous) insect herbivory occurred on the native congeners (*E. axillaris* and *E. foetida*) than on the nonnative invasive *E. uniflora* (Liu *et al.* 2007), at a time when the nonnative weevil was not well-established (Stricker and Stiling 2012). This example may not be the norm. A meta-analysis of paired invasive and native congeners revealed significantly higher insect herbivory on the paired native congeners, suggesting that enemy release of above-ground herbivores on nonnative plant species may facilitate their invasion (Liu and Stiling 2006).

### Limitations of ‘black box’ approach

We used a ‘black box’ approach, where we added fresh, field-collected soils to sterile soils for an inoculum of soil biota to the pots, and compared plant responses in these pots to those where the field-collected soils were sterilized before adding them to the pots. Thus, our research approach will only indicate the net effect of soil microbial communities on these plant species, and not which group of soil biota (symbionts and decomposers with positive effects or pathogens with negative effects) benefited from the experimental treatments. Additionally, by pooling the soil inoculum treatment within each site, the variation in plant–microbe interactions was decreased, as soil mixing causes the microbial composition across the site treatment to be homogenized (Reinhart and Rinella 2016; Rinella and Reinhart 2017). For example, if a pathogen influencing the invasive species was present in only a small fraction of our samples, soil mixing could have caused the pathogen to be included in all of the replicates (Rinella and Reinhart 2018). Future work on these species could characterize the microbial communities found in the soils of the sites and pots, as well as fungal and bacterial colonization of roots, to evaluate which groups of soil biota occurred in and benefitted from the treatments.

## Conclusion

The results from this experimental approach uniquely informs our understanding of the mechanisms that mediate range expansion of native and nonnative species under climate change. Plant–soil microbial interactions appeared as a form of biotic resistance to our nonnative study species, *E. uniflora*, as evidenced by the negative plant–microbe interactions found across its current and future range, as well as under different temperature conditions. *E. uniflora* had extremely high germination success and growth across all soil and temperature treatments, underscoring the invasive potential of this nonnative species. Soil microbes benefited our two native species differently across the current and future range. Outside of their current range and grown under future spring temperatures, *E. foetida* benefitted the most from soil microbes relative to *E. axillaris*, whereas the opposite was true in their current range of south Florida. This could have been due to the ability of this species to form new or greater positive generalist associations with soil biota, an escape from species-specific soil enemies, or variation between species in plant–soil microbe interactions by geographic region (McGinn *et al.* 2018).

The exact nature of environmental conditions associated with climatic change that nonnatives will be able to capitalize on, when compared with native species, are poorly understood (von Holle *et al.* 2010; Willis *et al.* 2010). While the role of species interactions is likely to be important in influencing range expansion by both native and nonnative species, few studies have focused on this (Svenning *et al.* 2014; Jones and Gilbert 2016). Specifically, the role of soil microbes on species range expansion has not received as much attention as that for its role in plant community membership (van Grunsven *et al.* 2007; Jones and Gilbert 2016). Our study demonstrates that soil microbiota negatively affect growth of one native and one nonnative species in habitats outside of their current range; however, one native species benefited more from soil microbes in the new range, and under warmer conditions, than the other native study species. Clearly more research is needed to elucidate the role of plant–soil microbe interactions on native and nonnative species, when compared with other factors, to understand the potential for biotic interactions to govern range expansion in a rapidly changing world.

## Supporting Information

The following supporting information is available in the online version of this article—

Table S1. *Eugenia uniflora* germination effect tests for climate (current, future), site (north, central and south) and soil (nonsterile, sterile).

Table S2a. Probability of germination effect tests for native *Eugenia* species (*axillaris, foetida*), site (north, central and south), and soil (nonsterile, sterile) at climate = current.

Table S2b Probability of germination effect tests for native *Eugenia* species (*axillaris, foetida*), site (north, central and south), and soil (nonsterile, sterile) at climate = future.

Table S3 Biomass effect tests for native *Eugenia* species (*axillaris, foetida*), site (north, central and south) and soil (nonsterile, sterile) at climate = current, conditional on germination.

plaa040_suppl_Supplementary_Material

## Data Availability

The data are available on the Environmental Data Initiative (https://portal.edirepository.org/nis/mapbrowse?scope=edi&identifier=585).

## Contributions by the Authors

B.V.H. conceived of the research questions and experimental design. B.V.H. wrote the manuscript, with contributions from S.E.W. and D.M.N. B.V.H. coordinated the student volunteers and managed the growth chamber experiment. S.E.W. set up and conducted the growth chamber experiment, along with other students. D.M.N. conducted the statistical analyses.

## Conflict of Interest

The authors declare that they have no conflict of interest.

## Notes

**Populations & Communities. Chief Editor**: Jean Burns
