# Supplementary material for: The influence of warming and biotic interactions on the potential for range expansion of native and nonnative species
Source: AoB Plants. 2020 Aug 18;12(5):plaa040. doi: 10.1093/aobpla/plaa040 (PMC7494242; doi:10.1093/aobpla/plaa040)
Supplement: plaa040_suppl_Supplementary_Material [file plaa040_suppl_supplementary_material.pdf]

Online Supporting Information

Table 1 - *Eugenia uniflora* germination effect tests for climate (current, future), site (north, central, and south) and soil (nonsterile, sterile).

Effect	Numerator df	Denominator df	F Value	p-value
climate	1	74	0.00	0.9967
site	2	74	0.00	1.0000
climate*site	2	74	0.00	1.0000
soil	1	74	0.00	0.9967
climate*soil	1	74	0.00	0.9992
site*soil	2	74	0.00	1.0000

Table 2a– Probability of germination effect tests for native *Eugenia* species (*axillaris, foetida*), site (north, central, and south), and soil (nonsterile, sterile) at climate = current.

Effect	Numerator df	Denominator df	F Value	p-value
Species	1	94	0.00	0.9553
Site	2	94	0.00	0.9989
Species*site	2	94	0.00	0.9994
Soil	1	94	0.00	0.9961
Species*soil	1	94	0.00	0.9971
Site*soil	2	94	0.00	0.9993
Species*site*soil	2	94	0.00	0.9992

Table 2b – Probability of germination effect tests for native *Eugenia* species (*axillaris, foetida*), site (north, central, and south), and soil (nonsterile, sterile) at climate = future.

Effect	Numerator df	Denominator df	F Value	p-value
Species	1	92	0.00	0.9543
Site	2	92	0.16	0.8550
Species*site	2	92	0.05	0.9475
Soil	1	92	0.00	0.9766
Species*soil	1	92	0.00	0.9757
Site*soil	2	92	0.01	0.9940
Species*site*soil	2	92	0.49	0.6115

Table 3 – Biomass effect tests for native *Eugenia* species (*axillaris*, *foetida*), site (north, central, and south) and soil (nonsterile, sterile) at climate = current, conditional on germination.

Effect	Numerator df	Denominator df	F Value	p-value
Species	1	35	1.72	0.1989
Site	2	35	1.23	0.3051
Species*site	2	35	0.88	0.4257
Soil	1	35	0.73	0.3986
Species*soil	1	35	0.43	0.5149
Site*soil	2	35	0.90	0.4166
